# Associations between health and sexual lifestyles in Britain: findings from the third National Survey of Sexual Attitudes and Lifestyles (Natsal-3)

**DOI:** 10.1016/S0140-6736(13)62222-9

**Published:** 2013-11-30

**Authors:** Nigel Field, Catherine H Mercer, Pam Sonnenberg, Clare Tanton, Soazig Clifton, Kirstin R Mitchell, Bob Erens, Wendy Macdowall, Frederick Wu, Jessica Datta, Kyle G Jones, Amy Stevens, Philip Prah, Andrew J Copas, Andrew Phelps, Kaye Wellings, Anne M Johnson

**Affiliations:** a Research Department of Infection and Population Health, University College London, London, UK; b NatCen Social Research, London, UK; c Department of Social and Environmental Health Research, London School of Hygiene and Tropical Medicine, London, UK; d Health Services Research and Policy, London School of Hygiene and Tropical Medicine, London, UK; e Centre for Endocrinology and Diabetes, Institute of Human Development, University of Manchester, Manchester, UK

## Abstract

**Background:**

Physical and mental health could greatly affect sexual activity and fulfilment, but the nature of associations at a population level is poorly understood. We used data from the third National Survey of Sexual Attitudes and Lifestyles (Natsal-3) to explore associations between health and sexual lifestyles in Britain (England, Scotland, and Wales).

**Methods:**

Men and women aged 16–74 years who were resident in households in Britain were interviewed between Sept 6, 2010, and Aug 31, 2012. Participants completed the survey in their own homes through computer-assisted face-to-face interviews and self-interview. We analysed data for self-reported health status, chronic conditions, and sexual lifestyles, weighted to account for unequal selection probabilities and non-response to correct for differences in sex, age group, and region according to 2011 Census figures.

**Findings:**

Interviews were done with 15 162 participants (6293 men, 8869 women). The proportion reporting recent sexual activity (one or more occasion of vaginal, oral, or anal sex with a partner of the opposite sex, or oral or anal sex or genital contact with a partner of the same sex in the past 4 weeks) decreased with age after the age of 45 years in men and after the age of 35 years in women, while the proportion in poorer health categories increased with age. Recent sexual activity was less common in participants reporting bad or very bad health than in those reporting very good health (men: 35·7% [95% CI 28·6–43·5] *vs* 74·8% [72·7–76·7]; women: 34·0% [28·6–39·9] *vs* 67·4% [65·4–69·3]), and this association remained after adjusting for age and relationship status (men: adjusted odds ratio [AOR] 0·29 [95% CI 0·19–0·44]; women: 0·43 [0·31–0·61]). Sexual satisfaction generally decreased with age, and was significantly lower in those reporting bad or very bad health than in those reporting very good health (men: 45·4% [38·4–52·7] *vs* 69·5% [67·3–71·6], AOR 0·51 [0·36–0·72]; women: 48·6% [42·9–54·3] *vs* 65·6% [63·6–67·4], AOR 0·69 [0·53–0·91]). In both sexes, reduced sexual activity and reduced satisfaction were associated with limiting disability and depressive symptoms, and reduced sexual activity was associated with chronic airways disease and difficulty walking up the stairs because of a health problem. 16·6% (95% CI 15·4–17·7) of men and 17·2% (16·3–18·2) of women reported that their health had affected their sex life in the past year, increasing to about 60% in those reporting bad or very bad health. 23·5% (20·3–26·9) of men and 18·4% (16·0–20·9) of women who reported that their health affected their sex life reported that they had sought clinical help (>80% from general practitioners; <10% from specialist services).

**Interpretation:**

Poor health is independently associated with decreased sexual activity and satisfaction at all ages in Britain. Many people in poor health report an effect on their sex life, but few seek clinical help. Sexual lifestyle advice should be a component of holistic health care for patients with chronic ill health.

**Funding:**

Grants from the UK Medical Research Council and the Wellcome Trust, with support from the Economic and Social Research Council and Department of Health.

## Introduction

Physical and mental health disorders, the drugs used to treat them, and long-term disability could greatly affect sexual lifestyles^1^, ^2^, ^3^, ^4^—ie, sexual activity, sexual behaviours, sexual problems, the formation and maintenance of relationships, and sexual satisfaction.^5^, ^6^, ^7^ For example, poor self-assessed general health is associated with reduced sexual activity and frequency of sexual activity in older people.^4^ However, most population-based studies investigating these associations have not been specifically designed to measure sexual behaviour, and have included only one or a few measures of health,^8^, ^9^, ^10^, ^11^, ^12^ surveyed only men,^13^, ^14^, ^15^ or been focused on older people.^4^, ^8^, ^13^, ^14^, ^16^ In the past 5 years, some studies have included both self-reported and biological or physical measures of a few specific disorders to explore associations with sexual function.^17^, ^18^ However, some chronic conditions—eg, arthritis, stroke, or heart disease—cannot easily be measured with biological or physical measures in large surveys of this kind. Therefore, the associations between ill health or disabilities and sexual lifestyles are not well described across the sexually active adult life at a population level. Moreover, the effect of health on people's sex lives is seldom considered in clinical practice,^19^, ^20^ and the evidence base that can be used to guide clinical management is small.

A decrease in sexual activity and frequency of sexual activity is associated with increasing age, although about half of people aged 65–74 years report sexual activity in the past year.^4^, ^5^, ^8^, ^9^, ^13^, ^21^ As the strongest driver of chronic ill health and long-term disability, age is therefore an important confounder in studies of the associations between health and sexual lifestyles. Likewise, the availability of partners—which could be limited by widowhood, no stable partner, or other reasons—affects these associations.^1^

In the past two decades, detailed information about sexual lifestyles across the population in Britain (England, Scotland, and Wales) has been obtained in the National Surveys of Sexual Attitudes and Lifestyles (Natsal). Howevever, these large probability sample surveys have not previously included detailed questions about health.^5^, ^6^, ^7^ In the third Natsal survey (Natsal-3), participants were asked to provide information about their health, including self-assessed general health, disability, functional impairment, and specific chronic conditions. Furthermore, people aged 16–74 years were included, which provides a rare opportunity to investigate the associations between sexual lifestyles and health across the life course in men and women.

We tested the hypothesis that poor health, disability, and specific chronic conditions would remain associated with three measures of sexual lifestyles after adjustment for age and relationship status: recent sexual activity, sexual satisfaction, and sexual response problems specific to men or women (erectile difficulties and vaginal dryness). To provide actionable information for clinicians and policy makers, we investigated whether people felt that any health condition or disability had affected their sex lives and the extent to which clinical advice had been sought.

## Methods

### Participants and procedures

Full details of the methods of Natsal-3 have been reported elsewhere.^5^, ^22^, ^23^ Briefly, we used a multistage, clustered, and stratified probability sample design. Men and women aged 16–74 years who were resident in households in Britain were interviewed between Sept 6, 2010, and Aug 31, 2012. An anonymised dataset will be deposited with the UK Data Archive, and the complete questionnaire and technical report will be available on the Natsal website on the day of publication.

Participants were interviewed in their own homes by professional interviewers without clinical qualifications using computer-assisted personal interview. Participants were asked about self-assessed general health with a widely cited and validated question,^24^ and about any longstanding and restricting illness or disability. To measure self-reported functional ability, participants were asked whether they had any difficulty walking up a flight of stairs because of a health problem.^25^ Body-mass index (BMI) was calculated from self-reported height and weight. To obtain information about self-reported clinical diagnoses of a range of chronic conditions, interviewers used show cards listing different disorders to ask participants whether they had any of the conditions listed. The first card asked whether a doctor had ever told them that they had cardiac or vascular diseases, hypertension, diabetes, chronic airways disease, and arthritis. Separate cards asked whether participants had ever had prostate diseases, broken hip or pelvis, and hip replacement, and whether they had received treatment from a health-care professional for any backache or bone or muscle disease lasting for more than 3 months in the past year.

Participants subsequently completed a computer-assisted self-interview, which included a validated two-question patient health questionnaire (PHQ-2), with which depressive symptoms in the past 2 weeks were assessed with two screening questions (scored 0–3).^26^ Participants were deemed to have depressive symptoms if they had a total score of 3 or more, a cutoff which has been previously validated.^27^ Participants were asked whether they had had any health condition or disability in the past year that they felt had affected their sexual activity or enjoyment in any way. Additionally, participants were asked whether they had sought help or advice about their sex life from a range of sources in the past year; more than one answer was allowed. The questionnaire underwent thorough cognitive testing and piloting, as previously reported.^23^, ^28^

The computer-assisted self-interview included many questions about sexual practices, partners, and activity.^22^ Here, we focus on four measures: recent sexual activity, satisfaction with sex life, sexual response problems, and relationship status. Recent sexual activity was defined as reporting of one or more occasion of vaginal, oral, or anal sex with a partner of the opposite sex, or oral or anal sex or genital contact with a partner of the same sex in the past 4 weeks. Participants were asked to think about their sex life in the past year in response to the statement “I feel satisfied with my sex life”. We deemed that those answering that they agreed or agreed strongly were satisfied with their sex life. Participants reporting at least one sexual partner in the past year were asked to report which, if any, of a range of sexual difficulties they had experienced for at least 6 months in the past year (the duration of symptoms corresponded to criteria in the 2013 *Diagnostic and Statistical Manual of Mental Disorders*, fifth edition^29^). Here, we focus on trouble achieving or maintaining an erection in men and an uncomfortably dry vagina in women, because these difficulties capture both physiological and psychological elements of sexual response and are associated with ageing.^30^ Finally, participants were divided into four categories on the basis of their relationship status at the time of interview: cohabiting with a partner (including marriage and civil partnerships); in a steady relationship (ie, expected to engage in sexual activity again with their partner) but not cohabiting with their partner; no steady relationship but reported previous cohabitation; and no steady relationship and never cohabited.

The Natsal-3 study was approved by the Oxfordshire Research Ethics Committee A (reference: 09/H0604/27). Participants provided oral informed consent for interviews.

### Statistical analysis

We did all analyses with the complex survey functions of Stata (version 12.1), accounting for stratification, clustering, and weighting of data. We included only participants who reported having had one or more sexual partner over the lifetime in our analysis, because those reporting no previous sexual experience did not complete the computer-assisted self-interview.

For sexual activity, satisfaction, response, and the perception of health effects on sex life, we report prevalences and 95% CIs in men and women by age group, relationship status, and each health variable. We weighted Natsal-3 data to adjust for the unequal probabilities of selection in terms of age and the number of adults in the eligible age range at an address. After application of these selection weights, the Natsal-3 sample was broadly representative of the British population compared with 2011 Census figures,^22^, ^31^ although men and London residents were slightly under-represented. Therefore, we also applied a non-response post-stratification weight to correct for differences in sex, age and Government Office Region between the achieved sample and the 2011 Census.

We used logistic regression models to calculate adjusted odds ratios (AORs) for associations with demographic and health variables, adjusting for age and relationship status. We additionally adjusted models investigating age and relationship status for self-assessed general health to understand the associations with age and relationship status independently of health status. We adjusted models investigating specific chronic health conditions for age, relationship status, and comorbidity (using categories of zero or one condition and more than one condition) to take account of the clustering of illnesses.

### Role of the funding source

The sponsors of the study had no role in study design, data collection, data analysis, data interpretation, or writing of the report. The corresponding author had full access to all the data in the study and had final responsibility for the decision to submit for publication.

## Results

Interviews were done with 15 162 participants (6293 men, 8869 women). 5994 men and 8452 women reported having had one or more sexual partner over the lifetime. The proportion of people in the worst self-reported health categories increased with age (table 1). The proportion of individuals reporting sexual activity in the past 4 weeks was highest in men aged 25–44 years and in women aged 25–34 years and decreased with age thereafter (table 2). The proportion of individuals reporting sexual activity in the past 4 weeks also varied by relationship status, with single people much less likely to report sexual activity than those in a steady relationship (table 2).

**Table 1:** General health characteristics of participants reporting at least one sexual partner over the lifetime, by age group and sex

		**16–24 years**	**25–34 years**	**35–44 years**	**45–54 years**	**55–64 years**	**65–74 years**	**All age groups**
**Men**								
Self-reported general health status								
	Very good	49·4% (46·6–52·2)	47·8% (45·0–50·6)	41·6% (37·9–45·4)	35·8% (40·6–48·8)	26·6% (38·2–46·3)	24·6% (37·7–46·2)	38·8% (37·4–40·3)
	Good	42·4% (39·6–45·1)	41·0% (38·2–43·9)	45·1% (41·3–48·9)	44·6% (40·6–48·8)	42·2% (38·2–46·3)	41·9% (37·7–46·2)	43·0% (41·4–44·6)
	Fair	7·5% (6·2–9·0)	9·9% (8·2–11·9)	10·9% (9·0–13·3)	15·2% (12·6–18·2)	21·7% (18·5–25·4)	27·1% (23·5–31·0)	14·4% (13·3–15·5)
	Bad or very bad	0·7% (0·4–1·2)	1·2% (0·8–1·9)	2·4% (1·6–3·6)	4·4% (3·2–6·0)	9·5% (7·4–12·1)	6·4% (4·6–8·9)	3·8% (3·3–4·4)
	Unweighted denominator	1689	1473	783	747	697	603	5992
	Weighted denominator	1210	1332	1386	1334	1093	779	7133
Longstanding illnesses or disability								
	None	86·2% (84·1–88·1)	82·6% (80·4–84·6)	76·2% (72·8–79·3)	63·8% (59·9–67·6)	46·6% (42·7–50·5)	43·2% (39·1–47·3)	68·6% (67·2–70·0)
	Non-limiting	8·3% (7·0–10·0)	8·4% (7·0–10·1)	10·6% (8·4–13·3)	18·2% (15·2–21·7)	24·4% (21·1–28·1)	27·3% (23·8–31·2)	15·2% (14·1–16·3)
	Limiting	5·4% (4·3–6·8)	9·0% (7·5–10·8)	13·2% (11·0–15·8)	17·9% (15·2–21·0)	29·0% (25·5–32·7)	29·5% (25·9–33·4)	16·2% (15·1–17·3)
	Unweighted denominator	1689	1472	783	748	697	603	5992
	Weighted denominator	1210	1332	1386	1335	1094	779	7135
Number of self-reported chronic conditions*								
	0	90·6% (89·0–92·1)	82·4% (80·0–84·6)	71·8% (68·1–75·2)	58·0% (54·1–61·8)	38·5% (34·7–42·4)	30·0% (26·1–34·1)	64·7% (63·2–66·2)
	1	8·5% (7·1–10·1)	14·5% (12·6–16·7)	21·4% (18·4–24·8)	28·3% (24·9–32·0)	33·0% (29·5–36·7)	33·8% (29·8–38·0)	22·3% (21·0–23·7)
	≥2	0·9% (0·6–1·4)	3·1% (2·1–4·6)	6·8% (5·3–8·7)	13·7% (11·2–16·6)	28·5% (25·2–32·1)	36·3% (32·3–40·5)	13·0% (11·9–14·0)
	Unweighted denominator	1687	1471	783	748	697	602	5988
	Weighted denominator	1208	1331	1386	1335	1094	778	7132
Difficulty walking up stairs because of a health problem								
	No difficulty	98·2% (97·4–98·8)	96·5% (95·0–97·5)	94·4% (92·5–95·8)	88·2% (85·5–90·5)	79·6% (76·3–82·7)	72·4% (68·3–76·2)	89·6% (88·6–90·5)
	Some difficulty	1·7% (1·1–2·5)	3·1% (2·1–4·6)	3·8% (2·6–5·5)	9·7% (7·7–12·2)	13·1% (10·6–16·0)	19·5% (16·3–23·1)	7·6% (6·8–8·4)
	Much difficulty or unable	0·1% (0·0–0·4)	0·4% (0·2–0·8)	1·8% (1·1–3·0)	2·1% (1·3–3·3)	7·3% (5·4–9·7)	8·1% (5·9–10·9)	2·8% (2·4–3·4)
	Unweighted denominator	1689	1473	782	748	698	603	5993
	Weighted denominator	1210	1332	1384	1335	1095	779	7135
**Women**								
Self-reported general health status								
	Very good	45·7% (43·4–48·1)	46·1% (43·8–48·3)	47·7% (44·6–50·9)	36·5% (39·1–45·7)	32·6% (36·5–43·0)	26·0% (37·6–44·8)	40·0% (38·8–41·2)
	Good	43·5% (41·1–45·8)	42·8% (40·6–45·1)	39·4% (36·4–42·6)	42·4% (39·1–45·7)	39·7% (36·5–43·0)	41·2% (37·6–44·8)	41·5% (40·3–42·7)
	Fair	8·9% (7·7–10·2)	9·5% (8·2–10·9)	10·0% (8·3–12·0)	15·5% (13·2–18·1)	19·6% (17·2–22·3)	23·6% (20·7–26·7)	13·9% (13·0–14·8)
	Bad or very bad	1·9% (1·4–2·7)	1·7% (1·2–2·3)	2·8% (2·0–4·0)	5·7% (4·4–7·2)	8·1% (6·4–10·2)	9·3% (7·3–11·8)	4·6% (4·1–5·2)
	Unweighted denominator	2081	2379	1158	1057	974	803	8452
	Weighted denominator	1172	1325	1393	1366	1166	856	7278
Longstanding illnesses or disability								
	None	82·6% (80·7–84·4)	78·7% (77·0–80·4)	73·6% (70·9–76·1)	63·7% (60·4–66·8)	51·4% (47·8–55·0)	41·4% (37·6–45·2)	66·8% (65·5–68·0)
	Non-limiting	8·5% (7·1–10·0)	11·0% (9·8–12·3)	12·8% (10·9–15·0)	12·9% (10·8–15·4)	20·2% (17·5–23·2)	26·2% (22·9–29·7)	14·5% (13·6–15·5)
	Limiting	8·9% (7·7–10·3)	10·3% (9·0–11·7)	13·7% (11·7–15·9)	23·4% (20·8–26·3)	28·4% (25·3–31·7)	32·5% (29·0–36·2)	18·7% (17·7–19·7)
	Unweighted denominator	2081	2378	1158	1057	974	803	8451
	Weighted denominator	1172	1324	1393	1366	1166	856	7278
Number of self-reported chronic conditions*								
	0	81·9% (80·0–83·6)	72·9% (70·9–74·9)	66·6% (63·7–69·4)	51·2% (47·8–54·7)	36·6% (33·3–40·1)	23·5% (20·5–26·8)	57·5% (56·2–58·7)
	1	15·8% (14·2–17·5)	21·3% (19·5–23·2)	23·0% (20·5–25·7)	28·6% (25·6–31·8)	31·9% (28·6–35·3)	30·9% (27·5–34·5)	24·9% (23·8–26·0)
	≥2	2·4% (1·8–3·2)	5·7% (4·8–6·8)	10·4% (8·7–12·3)	20·2% (17·7–22·9)	31·5% (28·4–34·8)	45·6% (41·9–49·4)	17·6% (16·6–18·6)
	Unweighted denominator	2080	2378	1156	1055	974	803	8446
	Weighted denominator	1171	1324	1391	1364	1166	856	7273
Difficulty walking up stairs because of health problem								
	No difficulty	96·4% (95·5–97·2)	95·7% (94·7–96·6)	92·0% (90·2–93·6)	82·5% (79·8–84·8)	74·5% (71·4–77·5)	61·4% (57·7–65·1)	85·2% (84·2–86·2)
	Some difficulty	3·2% (2·5–4·1)	3·4% (2·6–4·3)	6·1% (4·7–7·7)	12·2% (10·2–14·6)	17·5% (15·1–20·2)	25·9% (22·8–29·2)	10·4% (9·6–11·3)
	Much difficulty or unable	0·4% (0·2–0·7)	0·9% (0·6–1·4)	1·9% (1·2–2·9)	5·3% (4·1–6·8)	7·9% (6·2–10·0)	12·7% (10·4–15·5)	4·3% (3·8–4·9)
	Unweighted denominator	2081	2379	1158	1057	973	803	8451
	Weighted denominator	1172	1325	1393	1366	1165	856	7276

Data in parentheses are 95% CIs. Denominators are all individuals reporting one or more sexual partner over the lifetime and vary across variables because of item non-response. For all variables and both sexes, the χ^2^ p value for association with age was <0·0001.

* Measure of comorbidity includes arthritis, heart attack, coronary heart disease, angina, other forms of heart disease, hypertension, stroke, diabetes, broken hip or pelvis bone or hip replacement ever, backache lasting longer than 3 months, any other muscle or bone disease lasting longer than 3 months, depression, cancer, and any thyroid condition treated in the past year.

**Table 2:** Reporting of sexual activity in the past 4 weeks in relation to demographic and health characteristics, by sex

			**Men**					**Women**				
			Percentage reporting sexual activity	AOR	p value	Unweighted denominator	Weighted denominator	Percentage reporting sexual activity	AOR	p value	Unweighted denominator	Weighted denominator
All			67·4% (66·0–68·8)	..	..	5994	7137	61·6% (60·4–62·9)	..	..	8452	7278
Demographic characteristics												
	Age group				<0·0001					<0·0001		
		16–24 years	58·2% (55·5–60·8)	1·00	..	1689	1210	63·4% (60·9–65·7)	1·00	..	2081	1172
		25–34 years	79·4% (77·1–81·5)	1·05 (0·82–1·35)	..	1473	1332	80·0% (78·1–81·7)	0·82 (0·65–1·03)	..	2379	1325
		35–44 years	79·4% (76·3–82·2)	0·62 (0·46–0·82)	..	783	1386	75·8% (73·1–78·4)	0·45 (0·34–0·58)	..	1158	1393
		45–54 years	74·9% (71·4–78·2)	0·53 (0·39–0·72)	..	748	1335	68·0% (64·9–70·9)	0·30 (0·23–0·39)	..	1057	1366
		55–64 years	58·8% (54·7–62·7)	0·24 (0·18–0·32)	..	698	1095	43·2% (39·7–46·6)	0·10 (0·08–0·13)	..	974	1166
		65–74 years	39·3% (35·2–43·6)	0·09 (0·07–0·13)	..	603	779	22·7% (19·6–26·1)	0·04 (0·03–0·06)	..	803	856
	Relationship status				<0·0001					<0·0001		
		Living with a partner	78·2% (76·5–79·9)	1·00	..	2938	4649	73·5% (71·9–75·1)	1·00	..	4329	4646
		In a steady relationship, not cohabiting	90·7% (88·2–92·7)	1·35 (0·98–1·84)	..	955	766	88·4% (86·3–90·3)	1·18 (0·93–1·49)	..	1355	784
		No steady relationship, previously cohabited	27·7% (24·2–31·4)	0·09 (0·07–0·12)	..	752	669	17·9% (15·9–20·1)	0·07 (0·05–0·08)	..	1560	1118
		No steady relationship, never cohabited	27·8% (25·1–30·7)	0·02 (0·02–0·03)	..	1297	1003	23·6% (20·8–26·6)	0·02 (0·01–0·02)	..	1162	697
General health												
	Self-reported general health status				<0·0001					<0·0001		
		Very good	74·8% (72·7–76·7)	1·00	..	2411	2771	67·4% (65·4–69·3)	1·00	..	3429	2912
		Good	68·0% (65·9–69·9)	0·73 (0·62–0·87)	..	2532	3067	62·7% (60·8–64·6)	0·92 (0·79–1·07)	..	3545	3020
		Fair	54·5% (50·4–58·5)	0·51 (0·40–0·66)	..	826	1025	50·9% (47·6–54·3)	0·81 (0·65–1·01)	..	1125	1010
		Bad or very bad	35·7% (28·6–43·5)	0·29 (0·19–0·44)	..	223	270	34·0% (28·6–39·9)	0·43 (0·31–0·61)	..	353	335
	Longstanding illnesses or disability				<0·0001					<0·0001		
		None	71·7% (70·1–73·2)	1·00	..	4269	4897	67·1% (65·7–68·6)	1·00	..	5856	4859
		Non-limiting	64·1% (60·3–67·7)	0·92 (0·74–1·14)	..	803	1084	55·3% (51·8–58·7)	0·86 (0·71–1·04)	..	1131	1058
		Limiting	52·6% (48·9–56·2)	0·62 (0·51–0·76)	..	920	1155	46·8% (43·8–49·9)	0·43 (0·38–0·50)	..	1464	1360
	Number of self-reported chronic conditions*				<0·0001					<0·0001		
		0	71·7% (70·1–73·2)	1·00	..	4140	4615	68·5% (66·9–70·0)	1·00	..	5196	4179
		1	66·0% (62·8–69·0)	0·91 (0·74–1·11)	..	1178	1593	60·3% (57·7–62·9)	0·96 (0·81–1·13)	..	1993	1813
		≥2	48·9% (44·6–53·2)	0·58 (0·46–0·74)	..	670	924	41·1% (38·0–44·3)	0·67 (0·55–0·81)	..	1257	1282
	Body-mass index				0·0134					0·0011		
		Normal: 18·5–25 kg/m^2^	67·3% (65·1–69·4)	1·00	..	2633	2820	67·0% (65·2–68·7)	1·00	..	3326	4020
		Underweight: <18·5 kg/m^2^	46·0% (35·9–56·5)	0·51 (0·28–0·92)	..	128	105	59·3% (53·2–65·2)	0·80 (0·53–1·22)	..	315	211
		Overweight: 25–30 kg/m^2^	70·8% (68·5–73·0)	1·02 (0·85–1·23)	..	2009	2660	59·9% (57·5–62·3)	0·87 (0·74–1·03)	..	2062	1918
		Obese: 30–35 kg/m^2^	67·5% (63·6–71·2)	0·87 (0·69–1·10)	..	711	965	55·6% (51·9–59·3)	0·78 (0·63–0·96)	..	937	879
		Obese: >35 kg/m^2^	56·3% (48·8–63·6)	0·60 (0·40–0·90)	..	263	353	48·4% (43·5–53·3)	0·57 (0·43–0·76)	..	535	484
	Difficulty walking up stairs because of health problem				<0·0001					0·0008		
		No difficulty	69·8% (68·4–71·2)	1·00	..	5432	6393	65·3% (64·0–66·7)	1·00	..	7385	6201
		Some difficulty	52·7% (47·2–58·1)	0·68 (0·53–0·89)	..	406	540	42·8% (38·8–47·0)	0·72 (0·57–0·90)	..	749	759
		Much difficulty or unable	32·5% (24·6–41·7)	0·34 (0·21–0·54)	..	155	202	34·1% (28·3–40·5)	0·56 (0·40–0·78)	..	317	316
Specific health conditions												
	Any cardiac or vascular disease†				0·4173					0·2769		
		No	68·7% (67·2–70·1)	1·00	..	5697	6738	62·7% (61·4–64·0)	1·00	..	8193	7013
		Yes	46·6% (40·4–52·9)	0·88 (0·63–1·21)	..	296	398	32·4% (26·3–39·2)	0·82 (0·57–1·17)	..	256	263
	Hypertension				0·4103					0·6093		
		No	69·2% (67·7–70·6)	1·00	..	5401	6257	64·2% (62·9–65·5)	1·00	..	7612	6365
		Yes	55·0% (50·6–59·3)	0·90 (0·70–1·16)	..	592	879	43·5% (39·9–47·2)	1·06 (0·85–1·31)	..	837	911
	Diabetes				0·0384					0·1933		
		No	68·5% (67·1–69·9)	1·00	..	5719	6746	62·6% (61·3–63·9)	1·00	..	8158	6973
		Yes	48·3% (41·7–55·0)	0·69 (0·49–0·98)	..	274	391	38·8% (32·9–45·1)	0·80 (0·56 −1·12)	..	291	303
	Chronic airways disease				0·0041					0·0017		
		No	67·9% (66·5–69·3)	1·00	..	5931	7052	62·0% (60·8–63·3)	1·00	..	8365	7191
		Yes	27·3% (16·0–42·4)	0·35 (0·17–0·71)	..	62	84	25·3% (16·8–36·3)	0·41 (0·23–0·71)	..	84	84
	Arthritis				0·4949					0·0230		
		No	69·0% (67·6–70·4)	1·00	..	5535	6490	65·5% (64·2–66·8)	1·00	..	7495	6248
		Yes	51·3% (46·4–56·3)	0·91 (0·68–1·20)	..	458	646	37·8% (34·2–41·5)	0·76 (0·60–0·96)	..	954	1028
	Broken hip or pelvis or hip replacement				0·8368					0·0886		
		No	67·7% (66·3–69·1)	1·00	..	5909	7020	62·2% (60·9–63·4)	1·00	..	8326	7146
		Yes	53·8% (41·8–65·4)	0·94 (0·51–1·74)	..	81	114	31·2% (22·5–41·3)	0·62 (0·35–1·08)	..	123	129
	Backache, or bone or muscle disease for >3 months in past year				0·1032					0·7451		
		No	67·7% (66·2–69·2)	1·00	..	5367	6286	63·4% (62·0–64·7)	1·00	..	7292	6195
		Yes	65·4% (61·1–69·4)	1·23 (0·96–1·58)	..	625	849	51·5% (48·2–54·9)	1·04 (0·84–1·28)	..	1158	1081
	Depressive symptoms‡				<0·0001					0·0178		
		No	70·2% (68·8–71·6)	1·00	..	5214	6331	63·6% (62·3–65·0)	1·00	..	7292	6374
		Yes	52·3% (47·5–57·0)	0·55 (0·43–0·72)	..	607	660	53·5% (50·0–57·0)	0·76 (0·61–0·95)	..	983	780
	Prostate disease or surgery				0·8643							
		No	68·0% (66·6–69·4)	1·00	..	5805	6873	..	..	..	..	..
		Yes	52·4% (44·3–60·3)	0·97 (0·66–1·42)	..	185	261	..	..	..	..	..
	Menopause§									0·0060		
		No	..	..	..	..	..	23·7% (21·6–25·9)	1·00	..	1007	1092
		Yes	..	..	..	..	..	32·8% (30·6–35·1)	0·61 (0·42–0·87)	..	1006	1418

Data in parentheses are 95% CIs. Sexual activity was defined as one or more occasion of vaginal, oral, or anal sex with a partner of the opposite sex, or oral or anal sex or genital contact with a partner of the same sex in the past 4 weeks. All models were adjusted for age and relationship status. Models investigating age and relationship status also adjusted for self–assessed general health status. Models investigating specific conditions were also adjusted for comorbidity, for which comorbidity was coded as 0=0–1 specific conditions and 1=≥2 specific conditions. AOR=adjusted odds ratio.

* Measure of comorbidity and includes arthritis, heart attack, coronary heart disease, angina, other forms of heart disease, hypertension, stroke, diabetes, broken hip or pelvis bone or hip replacement ever, backache lasting longer than 3 months, any other muscle or bone disease lasting longer than 3 months, depression, cancer, and any thyroid condition treated in the past year.

† Heart attack, coronary heart disease, angina, other forms of heart disease, and stroke.

‡ Respondents were asked whether they had often been bothered by feeling down, depressed, or hopeless in the past 2 weeks, and whether they had often been bothered by little interest or pleasure in doing things in the past 2 weeks, with a validated two-question patient health questionnaire (PHQ-2).

§ Women deemed to be postmenopausal when they had not menstruated in the past year, with analysis restricted to those aged 45–64 years.

Overall, the proportion of men and women reporting sexual activity in the past 4 weeks was lower in those reporting bad or very bad health than in those reporting very good health, and this association remained after adjustment for age and relationship status (table 2). We recorded significant differences in the proportion reporting sexual activity in the past 4 weeks by health status in men aged 35–44 years and older, and in women aged 45–64 years (figure).

**Figure:**
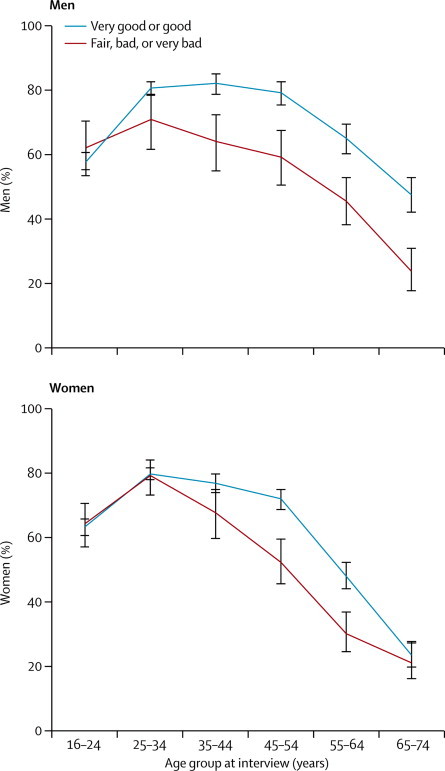
Reporting of sexual activity in past 4 weeks by sex and self-assessed general health status Bars show 95% CIs. Sexual activity was defined as one or more occasion of vaginal, oral, or anal sex with a partner of the opposite sex, or oral or anal sex or genital contact with a partner of the same sex in the past 4 weeks.

Similarly, we recorded strong associations between other broad measures of self-reported health and sexual activity in the past 4 weeks. After adjustment for age and relationship status, men and women reporting a limiting longstanding illness, two or more chronic conditions, a BMI of more than 35 kg/m^2^, or difficulty walking up stairs because of a health problem were significantly less likely to report sexual activity in the past 4 weeks than were those who did not report these issues (table 2). Although fewer individuals reporting specific health conditions reported sexual activity in the past 4 weeks than did those without these disorders, after adjustment for age, relationship status, and comorbidities, the associations were significant only for men and women with chronic airways disease or depressive symptoms, men with diabetes, and women with arthritis or who were postmenopausal (table 2). In sensitivity analyses, the patterns of association were broadly in the same direction and of a similar magnitude for reporting of sexual activity in the past 6 months and when the analysis was restricted to individuals aged 45–74 (data not shown).

More than 60% of men and women reported being satisfied with their sex life (table 3). Overall, after adjustment for relationship status and health, satisfaction decreased significantly after the age of 35 years in men and after the age of 25 years in women (table 3), but the association with age was weaker than for sexual activity. The percentage of men reporting sexual satisfaction was significantly lower in those reporting good, fair, or bad or very bad health than in those reporting very good health (table 3). The percentage of women reporting sexual satisfaction was also significantly lower in those reporting fair, bad, or very bad health than in those reporting very good health (table 3). Similar associations were recorded for other broad measures of health (table 3). However, after adjustment for age, relationship status, and comorbidities, depressive symptoms was the only specific health condition to be significantly associated with low sexual satisfaction (table 3).

**Table 3:** Reporting of satisfaction with sex life in the past year in relation to demographic and health characteristics, by sex

			**Men**					**Women**				
			Percentage reporting sexual satisfaction	AOR	p value	Unweighted denominator	Weighted denominator	Percentage reporting sexual satisfaction	AOR	p value	Unweighted denominator	Weighted denominator
All			61·7% (60·3–63·1)	..	..	5933	7110	61·4% (60·2–62·7)	..	..	8428	7278
Demographic characteristics												
	Age group				<0·0001					<0·0001		
		16–24 years	64·8% (62·2–67·3)	1·00	..	1601	1150	68·3% (66·0–70·5)	1·00	..	1985	1109
		25–34 years	68·7% (66·1–71·2)	0·88 (0·72–1·08)	..	1477	1332	68·5% (66·4–70·6)	0·70 (0·59–0·83)	..	2409	1334
		35–44 years	63·1% (59·4–66·7)	0·62 (0·49–0·79)	..	788	1390	64·0% (61·0–66·9)	0·53 (0·43–0·64)	..	1179	1416
		45–54 years	61·2% (57·2–65·0)	0·61 (0·47–0·78)	..	759	1355	59·8% (56·5–63·0)	0·46 (0·38–0·56)	..	1085	1395
		55–64 years	54·3% (50·2–58·3)	0·50 (0·39–0·64)	..	707	1106	54·8% (51·5–58·1)	0·41 (0·33–0·50)	..	977	1174
		65–74 years	53·9% (49·5–58·2)	0·51 (0·39–0·66)	..	601	778	48·9% (45·1–52·8)	0·38 (0·30–0·47)	..	793	849
	Relationship status				<0·0001					<0·0001		
		Living with a partner	64·9% (63·0–66·8)	1·00	..	2957	4685	67·0% (65·4–68·6)	1·00	..	4377	4709
		In a steady relationship, not cohabiting	83·0% (80·0–85·6)	2·04 (1·62–2·56)	..	959	768	79·6% (76·8–82·1)	1·47 (1·22–1·78)	..	1375	797
		No steady relationship, previously cohabited	38·5% (34·8–42·3)	0·37 (0·30–0·44)	..	791	704	34·9% (32·3–37·6)	0·29 (0·25–0·34)	..	1599	1131
		No steady relationship, never cohabited	45·7% (42·7–48·8)	0·31 (0·26–0·37)	..	1196	929	43·7% (40·5–47·0)	0·25 (0·21–0·30)	..	1042	616
General health												
	Self-reported general health status				<0·0001					0·0018		
		Very good	69·5% (67·3–71·6)	1·00	..	2382	2752	65·6% (63·6–67·4)	1·00	..	3408	2902
		Good	60·1% (57·8–62·3)	0·69 (0·59–0·80)	..	2502	3063	61·7% (59·7–63·6)	0·90 (0·79–1·01)	..	3533	3022
		Fair	50·0% (45·8–54·1)	0·52 (0·42–0·65)	..	816	1013	53·1% (49·8–56·4)	0·75 (0·63–0·89)	..	1132	1016
		Bad or very bad	45·4% (38·4–52·7)	0·51 (0·36–0·72)	..	231	280	48·6% (42·9–54·3)	0·69 (0·53–0·91)	..	355	338
	Longstanding illnesses or disability				0·0052					<0·0001		
		None	64·6% (63·0–66·3)	1·00	..	4209	4865	64·8% (63·3–66·3)	1·00	..	5828	4840
		Non-limiting	58·2% (53·9–62·3)	0·88 (0·72–1·08)	..	798	1085	57·7% (54·4–60·9)	0·86 (0·74–1·01)	..	1130	1057
		Limiting	52·5% (48·8–56·2)	0·77 (0·64–0·92)	..	924	1159	52·3% (49·4–55·1)	0·59 (0·52–0·68)	..	1469	1381
	Number of self-reported chronic conditions*				0·0075					<0·0001		
		0	65·1% (63·4–66·7)	1·00	..	4068	4577	67·0% (65·5–68·6)	1·00	..	5141	4142
		1	58·2% (55·0–61·3)	0·84 (0·71–1·00)	..	1179	1599	58·1% (55·6–60·6)	0·77 (0·67–0·88)	..	2020	1841
		≥2	51·0% (46·8–55·3)	0·74 (0·60–0·92)	..	679	929	48·1% (45·1–51·2)	0·62 (0·52–0·73)	..	1262	1291
	Body-mass index				0·0045					0·1190		
		Normal: 18·5–25 kg/m^2^	63·6% (61·4–65·8)	1·00	..	2599	2800	63·7% (61·9–65·5)	1·00	..	3329	4013
		Underweight: <18·5 kg/m^2^	58·2% (48·1–67·6)	0·94 (0·61–1·44)	..	120	100	65·7% (59·1–71·7)	1·20 (0·88–1·64)	..	302	200
		Overweight: 25–30 kg/m^2^	61·3% (58·9–63·6)	0·90 (0·77–1·04)	..	2007	2669	60·9% (58·5–63·3)	0·98 (0·86–1·12)	..	2084	1941
		Obese: 30–35 kg/m^2^	61·7% (57·6–65·7)	0·95 (0·77–1·17)	..	714	967	56·2% (52·7–59·8)	0·86 (0·72–1·02)	..	935	879
		Obese: >35 kg/m^2^	46·4% (39·5–53·6)	0·53 (0·38–0·74)	..	259	347	54·3% (49·4–59·2)	0·81 (0·64–1·02)	..	534	483
	Difficulty walking up stairs because of health problem				0·0119					0·0873		
		No difficulty	62·8% (61·3–64·3)	1·00	..	5366	6361	63·1% (61·7–64·4)	1·00	..	7361	6197
		Some difficulty	56·1% (50·5–61·6)	1·01 (0·78–1·30)	..	413	550	53·8% (49·8–57·7)	0·94 (0·78–1·14)	..	749	764
		Much difficulty or unable	42·2% (33·8–51·0)	0·61 (0·42–0·90)	..	153	198	47·6% (41·3–54·0)	0·77 (0·58–1·04)	..	317	316
Specific health conditions												
	Any cardiac or vascular disease†				0·7804					0·3598		
		No	62·3% (60·9–63·7)	1·00	..	5630	6706	61·9% (60·6–63·2)	1·00	..	8174	7015
		Yes	51·4% (45·2–57·5)	1·04 (0·77 – 1·41)	..	302	404	49·3% (42·4–56·2)	1·17 (0·84–1·64)	..	251	261
	Hypertension				0·3162					0·2841		
		No	62·9% (61·5–64·4)	1·00	..	5334	6226	62·7% (61·3–64·0)	1·00	..	7581	6358
		Yes	52·8% (48·4–57·2)	0·89 (0·71 – 1·12)	..	598	884	52·7% (49·0–56·3)	1·11 (0·92–1·35)	..	844	918
	Diabetes				0·4124					0·3036		
		No	62·3% (60·8–63·7)	1·00	..	5661	6719	62·1% (60·8–63·4)	1·00	..	8134	6968
		Yes	51·7% (44·7–58·7)	0·87 (0·63 – 1·21)	..	271	390	46·1% (39·8–52·5)	0·85 (0·62–1·16)	..	291	307
	Chronic airways disease				0·5281					0·1941		
		No	61·8% (60·3–63·2)	1·00	..	5868	7026	61·6% (60·4–62·9)	1·00	..	8341	7191
		Yes	55·2% (41·8–67·9)	1·22 (0·66 – 2·23)	..	64	84	42·1% (31·2–53·8)	0·72 (0·44 – 1·18)	..	84	85
	Arthritis				0·2272					0·4645		
		No	62·3% (60·8–63·7)	1·00	..	5472	6464	63·5% (62·2–64·8)	1·00	..	7476	6247
		Yes	55·6% (50·5–60·6)	1·18 (0·90 – 1·54)	..	460	646	48·9% (45·5–52·4)	0·93 (0·75–1·14)	..	949	1029
	Broken hip or pelvis or hip replacement				0·4519					0·1319		
		No	61·7% (60·3–63·1)	1·00	..	5847	6994	61·8% (60·5–63·0)	1·00	..	8301	7143
		Yes	59·3% (47·0–70·5)	1·24 (0·71 – 2·14)	..	81	112	41·5% (32·4–51·3)	0·72 (0·46–1·11)	..	125	134
	Backache, or bone or muscle disease for >3 months in past year				0·7165					0·0782		
		No	62·1% (60·6–63·6)	1·00	..	5301	6256	63·1% (61·7–64·4)	1·00	..	7252	6179
		Yes	58·7% (54·3–63·0)	1·04 (0·84 – 1·30)	..	630	853	52·1% (48·9–55·4)	0·85 (0·70–1·02)	..	1174	1079
	Depressive symptoms‡				<0·0001					<0·0001		
		No	63·8% (62·4–65·3)	1·00	..	5303	6430	64·0% (62·7–65·3)	1·00	..	7400	6466
		Yes	40·6% (36·1–45·3)	0·42 (0·33 – 0·52)	..	621	667	40·6% (37·1–44·2)	0·41 (0·34–0·49)	..	1020	804
	Prostate disease or surgery				0·3774							
		No	62·1% (60·7–63·6)	1·00	..	5737	6837	..	..	..	..-	..
		Yes	49·9% (42·4–57·5)	0·85 (0·60 – 1·21)	..	191	269	..	..	..	..	..
	Menopause§									0·7000		
		No	..	..	..	..	..	19·6% (17·7–21·7)	1·00	..	644	831
		Yes	..	..	..	..	..	37·8% (35·4–40·2)	1·05 (0·79–1·40)	..	1339	1714
Sexual activity										..		
	Sexually active in the past 4 weeks				<0·0001					<0·0001		
		No	37·1% (34·8–39·6)	1·00	..	1995	2174	38·0% (35·9–40·0)	1·00	..	3035	2645
		Yes	73·7% (71·9–75·4)	3·69 (3·13–4·35)	..	3801	4789	75·9% (74·5–77·3)	3·73 (3·22–4·32)	..	5184	4465

Data in parentheses are 95% CIs. All models were adjusted for age and relationship status. Models investigating age and relationship status also adjusted for self-assessed general health status. Models investigating specific conditions were also adjusted for comorbidity, for which comorbidity was coded as 0=0–1 specific conditions and 1=≥2 specific conditions. AOR=adjusted odds ratio.

* Measure of comorbidity and includes arthritis, heart attack, coronary heart disease, angina, other forms of heart disease, hypertension, stroke, diabetes, broken hip or pelvis bone or hip replacement ever, backache lasting longer than 3 months, any other muscle or bone disease lasting longer than 3 months, depression, cancer, and any thyroid condition treated in the past year.

† Heart attack, coronary heart disease, angina, other forms of heart disease, and stroke.

‡ Respondents were asked whether they had often been bothered by feeling down, depressed, or hopeless in the past 2 weeks, and whether they had often been bothered by little interest or pleasure in doing things in the past 2 weeks, with a validated two-question patient health questionnaire (PHQ-2).

§ Women deemed to be postmenopausal when they had not menstruated in the past year, with analysis restricted to those aged 45–64 years.

The percentage of individuals reporting sexual response problems increased with age: erectile difficulties were most common in men aged 65–74 years (affecting 26·2%, 95% CI 21·5–31·6) and vaginal dryness was most common in women aged 55–64 years (affecting 22·1%, 18·7–25·9; appendix). After adjustment for age and relationship status, participants with poorer general health, a limiting disability, or two or more chronic conditions were more likely than those who did not report these issues to have erectile difficulties or vaginal dryness (appendix). For specific health conditions in men, reporting of erectile difficulties was associated only with depressive symptoms after adjustment for age, relationship status, and comorbidities. In women, reporting of vaginal dryness was associated with a previous broken hip or pelvis or a hip replacement, depressive symptoms, being postmenopausal, and backache or bone or muscle disease for more than 3 months in the past year (appendix).

The percentage of male participants reporting that a health condition had affected their sex life in the past year increased in each successive age group (table 4). In women, the pattern was different: the percentage was highest in those aged 45–54 years, and lowest in those aged 16–24 years and 65–74 years (table 5). After adjustment for age and relationship status, we recorded strong associations in men and women between reporting that a health condition affected one's sex life and poorer self-assessed general health, limiting disabilities, one or more comorbidity, and difficulty with walking up stairs (Table 4, Table 5). By contrast with the small number of specific chronic conditions associated with sexual activity, satisfaction, and sexual response problems, reporting of an effect of health conditions on one's sex life was associated with a range of chronic health conditions: cardiac or vascular disease, chronic airways disease, backache, and depressive symptoms in both men and women; diabetes and prostate disease in men; and arthritis and hip or pelvis fracture or hip replacement in women (Table 4, Table 5).

**Table 4:** Reporting of health conditions affecting sexual activity or enjoyment in the past year and whether clinical advice has been sought, in relation to demographic and health characteristics of men

			**All men**					**Men whose health affects sex life**		
			Percentage reporting that their health affects sex life	Adjusted odds ratio	p value	Unweighted denominator	Weighted denominator	Percentage reporting that they sought clinical advice*	Unweighted denominator	Weighted denominator
All			16·6% (15·4–17·7)	..	..	5621	6870	23·5% (20·3–26·9)	845	1134
Demographic characteristics										
	Age group				<0·0001					
		16–24 years	6·1% (4·9–7·7)	1·00	..	1362	995	11·3% (5·7–21·2)	87	61
		25–34 years	10·6% (9·1–12·3)	1·64 (1·20–2·24)	..	1445	1296	15·9% (10·7–23·1)	161	137
		35–44 years	14·2% (11·7–17·0)	2·10 (1·47–3·01)	..	782	1378	23·6% (16·1–33·1)	121	195
		45–54 years	18·1% (15·2–21·4)	2·48 (1·76–3·49)	..	749	1343	25·2% (17·8–34·4)	139	240
		55–64 years	25·4% (22·0–29·0)	3·03 (2·17–4·21)	..	698	1095	25·5% (19·2–33·1)	178	278
		65–74 years	29·2% (25·2–33·5)	3·83 (2·73–5·38)	..	585	762	26·9% (20·3–34·6)	159	222
	Relationship status				0·4449					
		Living with a partner	17·5% (16·0–19·1)	1·00	..	2944	4665	23·5% (19·6–27·8)	496	813
		In a steady relationship, not cohabiting	12·6% (10·2–15·4)	1·05 (0·80–1·39)	..	955	766	34·6% (24·2–46·8)	109	96
		No steady relationship, previously cohabited	21·5% (18·4–25·0)	0·86 (0·67–1·11)	..	796	709	22·0% (15·0–31·1)	159	152
		No steady relationship, never cohabited	9·9% (7·8–12·5)	0·84 (0·62–1·14)	..	920	725	11·4% (5·7–21·5)	81	72
General health										
	Self-reported health status				<0·0001					
		Very good	7·6% (6·4–9·1)	1·00	..	2241	2638	21·7% (15·1–30·3)	154	201
		Good	14·6% (13·0–16·3)	1·88 (1·49–2·38)	..	2367	2963	22·7% (17·8–28·6)	311	432
		Fair	33·8% (30·2–37·6)	5·05 (3·90–6·54)	..	787	993	24·9% (19·2–31·6)	250	332
		Bad or very bad	60·7% (53·0–67·9)	14·57 (9·86–21·52)	..	224	273	24·8% (17·4–34·0)	129	166
	Longstanding illness or disability				<0·0001					
		None	9·0% (8·0–10·2)	1·00	..	3957	4669	20·5% (15·7–26·4)	319	421
		Non-limiting	21·2% (18·1–24·7)	2·19 (1·70–2·81)	..	765	1061	26·0% (18·9–34·6)	154	225
		Limiting	43·1% (39·4–46·9)	6·22 (4·99–7·76)	..	897	1139	24·8% (20·3–29·9)	372	488
	Number of self-reported chronic conditions†				<0·0001					
		0	8·3% (7·3–9·4)	1·00	..	3812	4381	17·7% (12·8–24·1)	297	364
		1	22·7% (20·2–25·5)	2·82 (2·26–3·52)	..	1123	1540	27·3% (21·5–34·1)	253	347
		≥2	44·6% (40·6–48·8)	7·16 (5·59–9·17)	..	686	948	25·2% (20·2–30·9)	295	423
	Body-mass index				<0·0001					
		Normal: 18·5–25 kg/m^2^	13·4% (11·8–15·1)	1·00	..	2428	2669	22·3% (17·2–28·3)	296	356
		Underweight: <18·5 kg/m^2^	12·3% (6·4–22·4)	1·28 (0·62–2·65)	..	95	84	33·0% (7·8–74·3)	11	10
		Overweight: 25–30 kg/m^2^	16·4% (14·6–18·4)	1·06 (0·86–1·30)	..	1948	2615	25·1% (20·0–31·1)	299	428
		Obese: 30–35 kg/m^2^	20·2% (17·1–23·8)	1·30 (1·01–1·68)	..	696	954	25·8% (18·4–34·9)	133	190
		Obese: >35 kg/m^2^	31·4% (25·4–38·2)	2·27 (1·62–3·17)	..	254	342	22·2% (13·5–34·2)	77	107
	Difficulty walking up stairs because of health problem				<0·0001					
		No difficulty	12·6% (11·5–13·8)	1·00	..	5069	6133	23·9% (20·0–28·3)	584	773
		Some difficulty	43·4% (38·0–49·1)	4·06 (3·13–5·28)	..	401	542	24·7% (18·3–32·5)	169	232
		Much difficulty or unable	65·6% (56·9–73·3)	9·80 (6·51–14·76)	..	150	193	17·7% (10·4–28·4)	91	127
Specific health conditions										
	Any cardiac or vascular disease‡				<0·0001					
		No	14·9% (13·8–16·0)	1·00	..	5376	6528	22·0% (18·7–25·7)	735	967
		Yes	48·8% (42·0–55·6)	2·14 (1·57–2·91)	..	244	341	31·9% (23·5–41·7)	110	166
	Hypertension				0·7823					
		No	14·1% (13·0–15·3)	1·00	..	5031	5994	23·6% (19·9–27·7)	652	843
		Yes	33·2% (29·1–37·6)	1·04 (0·81–1·32)	..	589	876	23·0% (17·4–29·8)	193	291
	Diabetes				0·0045					
		No	15·1% (14·0–16·3)	1·00	..	5357	6486	22·6% (19·2–26·3)	732	976
		Yes	41·1% (34·7–47·9)	1·55 (1·15–2·09)	..	263	383	28·8% (20·3–39·2)	113	158
	Chronic airways disease				<0·0001					
		No	16·1% (15·0–17·2)	1·00	..	5558	6787	23·8% (20·6–27·4)	812	1087
		Yes	56·5% (42·0–69·9)	4·12 (2·09–8·12)	..	62	83	15·1% (6·1–32·8)	33	47
	Arthritis				0·5600					
		No	14·8% (13·6–16·0)	1·00	..	5172	6235	24·1% (20·6–28·0)	694	917
		Yes	34·1% (29·4–39·2)	1·08 (0·83–1·41)	..	448	635	20·6% (14·4–28·5)	151	217
	Broken hip or pelvis or hip replacement				0·1012					
		No	16·1% (15·0–17·3)	1·00		5536	6754	23·2% (20·0–26·8)	813	1086
		Yes	41·4% (29·9–53·9)	1·55 (0·92–2·61)	..	81	112	29·9% (15·9–49·0)	31	46
	Backache, or bone or muscle disease for >3 months in past year				0·0277					
		No	14·2% (13·1–15·4)	1·00	..	5013	6035	24·9% (21·1–29·0)	649	855
		Yes	33·3% (29·3–37·5)	1·31 (1·03–1·67)	..	606	834	19·1% (13·8–25·9)	195	278
	Depressive symptoms§				<0·0001					
		No	14·8% (13·7–16·0)	1·00	..	5027	6215	22·6% (19·1–26·5)	664	916
		Yes	33·7% (29·4–38·4)	2·62 (2·05–3·34)	..	582	638	26·6% (19·8–34·8)	179	215
	Prostate disease or surgery				0·0026					
		No	15·7% (14·6–16·9)	1·00	..	5427	6598	22·6% (19·4–26·3)	768	1032
		Yes	37·6% (30·3–45·5)	1·82 (1·23–2·68)	..	190	269	32·0% (22·0–43·9)	76	101
Sexual activity										
	Sexually active in the past 4 weeks				0·0275					
		No	21·2% (19·0–23·5)	1·00	..	1704	1952	20·3% (15·7–25·9)	314	413
		Yes	14·3% (13·0–15·7)	0·80 (0·65–0·97)	..	3780	4769	25·1% (21·0–29·7)	492	682
	Satisfied with sex life				<0·0001					
		No	23·6% (21·5–25·8)	1·00	..	2099	2586	27·4% (23·1–32·3)	458	610
		Yes	12·3% (11·0–13·6)	0·50 (0·42–0·59)	..	3510	4269	18·8% (14·7–23·8)	387	524

Data in parentheses are 95% CIs. All models were adjusted for age and relationship status. Models investigating age and relationship status also adjusted for self-assessed general health status. Models investigating specific conditions were also adjusted for comorbidity, for which comorbidity was coded as 0=0–1 specific conditions and 1=≥2 specific conditions.

* General practitioner, sexual health clinic, other clinics or doctors, or psychiatrist or psychologist.

† Measure of comorbidity and includes arthritis, heart attack, coronary heart disease, angina, other forms of heart disease, hypertension, stroke, diabetes, broken hip or pelvis bone or hip replacement ever, backache lasting longer than 3 months, any other muscle or bone disease lasting longer than 3 months, depression, cancer, and any thyroid condition treated in the past year.

‡ Heart attack, coronary heart disease, angina, other forms of heart disease, and stroke.

§ Respondents were asked whether they had often been bothered by feeling down, depressed, or hopeless in the past 2 weeks, and whether they had often been bothered by little interest or pleasure in doing things in the past 2 weeks, with a validated two-question patient health questionnaire (PHQ-2).

**Table 5:** Reporting of health conditions affecting sexual activity or enjoyment in the past year and whether clinical advice has been sought, in relation to demographic and health characteristics of women

			**All women**					**Women whose health affects sex life**		
			Percentage reporting that their health affects sex life	Adjusted odds ratio	p value	Unweighted denominator	Weighted denominator	Percentage reporting that they sought clinical advice*	Unweighted denominator	Weighted denominator
All			17·2% (16·3–18·2)	..	..	8097	7071	18·4% (16·0–20·9)	1322	1215
Demographic characteristics										
	Age group				<0·0001					
		16–24 years	13·2% (11·5–15·2)	1·00	..	1716	957	24·4% (18·7–31·2)	223	126
		25–34 years	18·5% (16·8–20·4)	1·27 (1·03–1·56)	..	2376	1312	19·9% (15·9–24·6)	425	243
		35–44 years	16·6% (14·4–19·0)	1·02 (0·80–1·29)	..	1174	1404	16·4% (11·7–22·4)	190	233
		45–54 years	19·6% (17·1–22·3)	1·03 (0·81–1·31)	..	1064	1374	16·8% (11·9–23·2)	218	269
		55–64 years	18·8% (16·2–21·7)	0·86 (0·66–1·12)	..	975	1175	22·5% (16·3–30·1)	165	219
		65–74 years	14·8% (12·1–17·9)	0·58 (0·43–0·77)	..	792	848	9·2% (4·8–16·9)	101	125
	Relationship status				<0·0001					
		Living with a partner	19·1% (17·8–20·4)	1·00	..	4357	4678	18·5% (15·6–21·7)	809	889
		In a steady relationship, not cohabiting	17·2% (14·9–19·8)	0·81 (0·66–1·00)	..	1373	796	19·2% (14·2–25·5)	220	137
		No steady relationship, previously cohabited	12·2% (10·5–14·0)	0·42 (0·35–0·52)	..	1620	1153	17·1% (12·5–22·9)	214	140
		No steady relationship, never cohabited	10·9% (8·5–14·0)	0·42 (0·32–0·57)	..	738	439	17·7% (10·2–29·0)	78	48
General health										
	Self-reported health status				<0·0001					
		Very good	9·6% (8·4–10·8)	1·00	..	3245	2799	21·4% (16·3–27·5)	299	267
		Good	15·3% (14·0–16·7)	1·80 (1·51–2·15)	..	3392	2928	18·0% (14·5–22·1)	516	448
		Fair	30·6% (27·7–33·8)	4·99 (4·05–6·15)	..	1110	1009	20·5% (15·9–26·0)	319	307
		Bad or very bad	57·5% (51·5–63·4)	17·29 (12·96–23·05)	..	350	335	11·7% (7·8–17·1)	188	193
	Longstanding illness or disability				<0·0001					
		None	11·3% (10·3–12·3)	1·00	..	5550	4666	18·7% (15·3–22·7)	621	525
		Non-limiting	14·3% (12·2–16·8)	1·48 (1·19–1·85)	..	1107	1042	25·2% (18·4–33·5)	155	149
		Limiting	39·8% (36·8–42·8)	6·29 (5·28–7·50)	..	1439	1363	16·2% (13·0–19·8)	546	540
	Number of self-reported chronic conditions†				<0·0001					
		0	10·6% (9·6–11·7)	1·00	..	4865	3976	18·2% (14·5–22·5)	510	421
		1	20·0% (18·0–22·2)	2·47 (2·06–2·96)	..	1953	1788	20·0% (16·0–24·7)	408	358
		≥2	33·5% (30·6–36·4)	6·26 (5·11–7·68)	..	1278	1306	17·2% (13·5–21·7)	404	435
	Body-mass index				0·0039					
		Normal: 18·5–25 kg/m^2^	16·5% (15·1–18·0)	1·00	..	3844	3220	20·0% (16·5–24·1)	601	532
		Underweight: <18·5 kg/m^2^	18·8% (13·8–25·1)	1·32 (0·89–1·95)	..	261	178	17·3% (7·4–35·4)	46	33
		Overweight: 25–30 kg/m^2^	15·9% (14·1–17·8)	0·93 (0·78–1·10)	..	2039	1906	14·2% (10·5–18·9)	311	302
		Obese: 30–35 kg/m^2^	20·4% (17·4–23·6)	1·28 (1·03–1·60)	..	920	874	22·3% (15·9–30·4)	178	176
		Obese: >35 kg/m^2^	22·0% (17·9–26·6)	1·43 (1·09–1·88)	..	529	482	19·3% (12·5–28·6)	110	105
	Difficulty walking up stairs because of health problem				<0·0001					
		No difficulty	13·8% (12·9–14·8)	1·00	..	7041	5995	20·2% (17·3–23·5)	949	827
		Some difficulty	30·1% (26·5–34·0)	3·34 (2·72–4·11)	..	742	760	19·6% (14·6–25·9)	225	227
		Much difficulty or unable	51·3% (44·8–57·7)	8·81 (6·65–11·68)	..	313	315	7·0% (4·0–12·1)	148	161
Specific health conditions										
	Any cardiac or vascular disease‡				0·0043					
		No	16·8% (15·8–17·8)	1·00	..	7900	6864	18·7% (16·3–21·3)	1269	1148
		Yes	32·1% (25·0–40·2)	1·71 (1·18–2·46)	..	195	205	12·4% (4·8–28·3)	52	66
	Hypertension				0·1057					
		No	16·5% (15·5–17·6)	1·00	..	7262	6161	18·9% (16·4–21·7)	1162	1018
		Yes	21·8% (18·7–25·2)	0·83 (0·67–1·04)	..	833	909	15·5% (10·0–23·1)	159	196
	Diabetes				0·0637					
		No	16·7% (15·7–17·7)	1·00	..	7808	6766	18·1% (15·7–20·7)	1243	1125
		Yes	29·2% (23·5–35·7)	1·35 (0·98–1·85)	..	287	304	21·8% (13·9–32·5)	78	89
	Chronic airways disease				0·0002					
		No	16·9% (16·0–17·9)	1·00	..	8009	6983	18·5% (16·2–21·2)	1292	1180
		Yes	38·9% (27·7–51·4)	2·69 (1·62–4·49)	..	86	86	11·0% (3·7–28·5)	29	34
	Arthritis				0·0177					
		No	15·6% (14·7–16·7)	1·00	..	7146	6038	19·2% (16·6–22·2)	1078	942
		Yes	26·4% (23·3–29·8)	1·29 (1·05–1·60)	..	949	1031	15·2% (10·9–20·8)	243	272
	Broken hip or pelvis or hip replacement				0·0069					
		No	16·9% (15·9–17·9)	1·00	..	7972	6939	18·9% (16·5–21·5)	1285	1168
		Yes	35·0% (25·8–45·4)	1·85 (1·19–2·88)	..	122	130	6·3% (1·9–18·6)	36	46
	Backache, or bone or muscle disease for >3 months in past year				<0·0001					
		No	14·5% (13·5–15·5)	1·00	..	6942	5988	18·6% (15·9–21·6)	962	867
		Yes	32·3% (29·3–35·5)	1·70 (1·40–2·06)	..	1153	1081	17·8% (13·7–22·8)	360	347
	Depressive symptoms§				<0·0001					
		No	14·9% (13·9–15·9)	1·00	..	7102	6278	17·7% (15·1–20·7)	1002	935
		Yes	35·8% (32·2–39·5)	2·75 (2·28–3·33)	..	985	781	20·6% (16·2–25·9)	320	279
	Menopause¶				0·2097					
		No	16·9% (14·0–20·3)	1·00	..	632	822	9·6% (4·8–18·2)	109	139
		Yes	20·3% (18·1–22·8)	1·25 (0·88–1·76)	..	1389	1705	23·1% (18·0–29·1)	270	345
Sexual activity										
	Sexually active in the past 4 weeks				0·0681					
		No	16·8% (15·2–18·5)	1·00	..	2736	2469	14·7% (11·3–18·9)	409	414
		Yes	17·2% (16·0–18·4)	0·85 (0·71–1·01)	..	5168	4449	20·6% (17·6–24·1)	866	760
	Satisfied with sex life				<0·0001					
		No	23·2% (21·5–25·0)	1·00	..	2999	2669	20·6% (17·3–24·3)	669	619
		Yes	13·5% (12·5–14·7)	0·45 (0·39–0·52)	..	5061	4365	16·0% (13·0–19·5)	650	591

Data in parentheses are 95% CIs. All models were adjusted for age and relationship status. Models investigating age and relationship status also adjusted for self-assessed general health status. Models investigating specific conditions were also adjusted for comorbidity, for which comorbidity was coded as 0=0–1 specific conditions and 1=≥2 specific conditions.

* General practitioner, sexual health clinic, other clinics or doctors, or psychiatrist or psychologist.

† Measure of comorbidity and includes arthritis, heart attack, coronary heart disease, angina, other forms of heart disease, hypertension, stroke, diabetes, broken hip or pelvis bone or hip replacement ever, backache lasting longer than 3 months, any other muscle or bone disease lasting longer than 3 months, depression, cancer, and any thyroid condition treated in the past year.

‡ Heart attack, coronary heart disease, angina, other forms of heart disease, and stroke.

§ Respondents were asked whether they had often been bothered by feeling down, depressed, or hopeless in the past 2 weeks, and whether they had often been bothered by little interest or pleasure in doing things in the past 2 weeks, with a validated two-question patient health questionnaire (PHQ-2).

¶ Women deemed to be postmenopausal when they had not menstruated in the past year, with analysis restricted to those aged 45–64 years.

Overall, 7·0% (95% CI 6·2–7·8) of men and 6·7% (6·1–7·4) of women reported having sought clinical help or advice about their sex life. Seeking clinical help was more common in individuals reporting that a health condition affected their sex lives than in those who did not (men: 23·5% [95% CI 20·3–26·9] *vs* 3·7% [3·2–4·4]; women: 18·4% [17·5–22·8] *vs* 4·3% [3·8–4·9]). Most participants who reported seeking help asked a family doctor for advice (men: 85·4%, 95% CI 78·7–90·3; women: 80·7%, 74·9–85·4), with smaller proportions reporting visiting a sexual health clinic (men: 7·6%, 4·5–12·5; women: 11·2%, 8·0–15·5), another type of clinic (men: 6·8%, 4·0–11·3; women: 8·8%, 5·5–13·7), or a psychiatrist or psychologist (men: 6·5%, 3·2–12·5; women: 11·3%, 7·6–16·4; data not shown). A higher proportion of men than women who had reported that their health affected their sex life reported that they had sought clinical advice (Table 4, Table 5). However, the proportions did not vary substantially or consistently by health status (Table 4, Table 5).

## Discussion

In our large, population-based study, we have shown that poorer physical health, limiting disabilities, functional impairment, and depressive symptoms are associated with decreased sexual activity and sexual satisfaction and increased reporting of sexual response problems (erectile difficulties and vaginal dryness) in men and women aged 16–74 years in Britain (panel). Furthermore, about 17% of all men and women reported that their health had affected their sex life in the past year. This proportion rose to roughly 60% in participants with bad or very bad health. Less than a quarter of men and a fifth of women who reported that their health affected their sex life had sought clinical help, which was usually from general practitioners rather than specialists. Overall, we have identified strong associations between health and sexual lifestyles, and established that many people are aware of an effect of ill health on their sex life.

Panel **Research in context**

**Systematic review**

A range of health conditions, disabilities, and drugs affects sexual health and lifestyles.^7^, ^11^ The effects are important throughout life, but could be particularly important in older age, when chronic ill health is most common. However, population-based studies in which these issues have been investigated have often had narrow age ranges, few measures of health, and inconsistent findings.^3^ Moreover, these issues are seldom discussed in clinical practice,^11^ and the evidence base to guide clinical management and self-help is small.

**Interpretation**

As far as we are aware, our analysis is the first in which the relationships between sexual lifestyles and health have been studied across a wide age range in men and women, and with a broad range of self-reported health measures. We have shown that health and sexual lifestyles are associated in most sexually active age groups, even after adjustment for age and relationship status. However, we have also shown that many people in poor health and at older ages report an active or satisfying sex life, or both. Many people reported that health conditions had had an effect on their sex lives in the past year—an effect that persisted into older age. This association was significantly higher in participants reporting ill health. However, of individuals reporting a health condition that affected their sex life, only a quarter of men and a fifth of women had sought clinical help or advice. Our findings should help clinicians, their patients, and policy makers to consider the continuation of sexual activity and enjoyment in the face of ill health. Practitioners should consider giving appropriate advice about sexual lifestyles to promote the overall wellbeing of patients with chronic conditions.

Our sample encompasses most of the sexually active lifespan, adding to previous work by emphasising the differences in reported sexual activity according to health status, including at younger ages. For example, the proportion of men aged 35–44 years who reported sexual activity in the past 4 weeks was much lower for those with fair, bad, or very bad health than for those with very good or good health, and similar to the proportion in men reporting good or very good health but 20 years older. We recorded a similar pattern in women, although we noted no difference by health status in the oldest women. This finding could be a result of the low prevalence of sexual activity in older women, and possibly related to their partnership status and their partners' age and health. An area of future research will be to investigate the role of partners' characteristics—eg, through studies of both partners in a relationship. Nevertheless, about a third of men and women in bad or very bad health reported sexual activity in the past 4 weeks, and just less than half of the same group reported satisfaction with their sex lives. Beckman and colleagues^21^ suggested that more accepting attitudes have contributed to increased reporting of sexual activity in older people; the message that poor health need not mean the end of an active or satisfying sex life is an important one.

Although we sought to minimise reporting bias by using computer-assisted self-interview for the most sensitive questions in addition to non-response weighting,^30^ our cross-sectional data should be interpreted with caution. We relied on self-reported diagnoses, which can lead to misclassification errors, although previous studies have shown good levels of agreement between self-reported chronic disease and medical records,^32^, ^33^ and self-report remains the primary method to assess health status in many large population surveys. Furthermore, the patterns of disease prevalence that we recorded by age and sex were similar to those reported in the Health Survey for England.^34^ Participants with undiagnosed or preclinical disease could not be identified in our study, which could have meant the effect size was underestimated. Our study did not include physical or biological measures of health, which can be used to validate self-reporting.^35^ Additionally, we were unable to account for specific drugs or partners' health status, or to explore severity within specific conditions, and we cannot attribute directional causality.

The Natsal-3 sample was broadly representative of the general population of Britain in terms of self-reported general health, marital status, and ethnic origin, on the basis of 2011 census data, after weighting for key sociodemographic characteristics.^22^, ^31^ However, the data are susceptible to participation biases—eg, poor health could affect willingness to participate—and the sampling frame did not include individuals in residential or nursing care. Although we have referred to the life course, our findings are not generalisable to people older than 74 years. Our findings at different ages could be partly affected by differences in sexual behaviour between generations.^5^ In the future, longitudinal studies and investigations including biological and physical measures of health will be important in the refinement of our understanding and establishment of causality. Nevertheless, the strength and novelty of our study lie in its size, population representativeness, and broad range of detailed sexual lifestyle measures. Importantly, we could explore the associations between sexual lifestyles and health, with measures of both broad health and specific conditions, independently of the key confounders of age and partnership status. This Article builds a compelling and consistent picture showing the strong associations between sexual lifestyles and health across the life course.

Although our findings are consistent with previous reports,^4^, ^8^, ^10^, ^13^, ^14^, ^36^ not all studies have reached the same conclusions about the associations with general health, particularly in women. A large Spanish cross-sectional survey of people older than 65 years^8^ showed that sexual activity was associated with self-assessed general health status in men but not women. A US study^11^ showed that poor physical health did not contribute to the age-related decrease in sexual frequency in women aged 44–72 years, although health did contribute in men. Additionally, another US study^37^ showed that ill health was unrelated to frequency of sexual activity in retired men and women older than 45 years. However, these studies were restricted in age range, and, by including a wide age range and using a large sample size, we were able to identify the associations with ill health separately from those with age. With adjustment for age and additionally for the availability of partners and comorbidities, we noted strong associations between sexual lifestyles and some specific chronic health conditions. Our data support other studies linking mental ill health and sexual problems.^8^, ^13^, ^14^ Diabetes was associated with reduced sexual activity in men but not women, but we note other studies showed associations only for women,^4^, ^17^ which could be a result of differences in methods used. In our study, hypertension was not associated with sexual activity, satisfaction, or sexual responses, although other studies—in some of which investigators used sphygmomanometry—have shown that sexual activity is reduced in women with hypertension,^18^ but not in men.^13^, ^18^

To our knowledge, no other large population survey of sexual lifestyles has investigated whether people felt that a health condition affected their sexual activity or enjoyment. Reporting of a health condition that affected an individual's sex life increased in each age group in men but not women. These findings are consistent with previous work suggesting that an impaired sexual response is less likely to be problematic for older women than younger women.^38^

Most people reporting a condition affecting their sex life in the past year had not sought clinical advice. When they had, they consulted general practitioners or family doctors and seldom specialists, which corroborates findings that general practitioners are often the first point of contact for sexual concerns.^39^ Evidence suggests that, although many major health conditions are recognised to affect sexual function,^40^ clinical advice about the effect of ill health on sexual activity is often not given, even when the condition is gynaecological or genital,^41^, ^42^ and doctors rarely ask patients about their sexual function.^43^ Not only are patients sometimes unwilling to discuss problems, but health-care professionals might also have poor awareness and little training about advising and communicating with patients about sexual problems.^44^ This issue needs to be addressed, because sexual problems are common and can cause distress,^30^ can indicate underlying physical or mental health problems, and can be caused iatrogenically (eg, by drugs or surgery).^45^ Our findings indicate that many patients with chronic ill health are already aware that their health has an effect on their sex lives. Therefore, awareness needs to increase, guidance improve, and communication skills built so that problems are acknowledged, ameliorated, and openly discussed in appropriate ways.

On the one hand, people are living longer and have expectations of continued sexual fulfilment as they age.^46^, ^47^ But on the other hand, ill health increases in old age; the proportion of the English population reporting one or more chronic conditions rises from 17% in individuals younger than 40 years to 60% in those older than 60 years.^48^ The sexual health of older people has been a neglected area of research.^44^, ^47^, ^49^ More recent public health policy, such as a WHO 2013 report,^50^ reflects an increasing awareness of the interdependence between sexual health and general health. A UK Department of Health 2013 report^51^ drew attention to the effects of long-term health conditions on erectile function and the effect of cancer on sexual health in older age. Our study suggests that strategic frameworks could go further and include associations between health—eg, disabilities or mental health—and sexual lifestyles at younger ages, as well as the broader associations between sexual lifestyles and chronic ill health.

Sexual lifestyles are strongly linked to overall health and wellbeing. The finding that many people who felt that their health affected their sex life seldom sought clinical help suggests an unmet clinical need that is inadequately recognised in present medical practice and health policy. Our data should help to challenge stereotypes and inform sexual policy for all ages.

## Supplementary Material

Supplementary appendix

Supplementary video Results of over 45,000 respondents, recording the dramatic changes in our sexual lifestyles over the past 60 years.
