# Supplementary material for: Associations between health and sexual lifestyles in Britain: findings from the third National Survey of Sexual Attitudes and Lifestyles (Natsal-3)
Source: Lancet. 2013 Nov 30;382(9907):1830–44. doi: 10.1016/S0140-6736(13)62222-9 (PMC3898988; doi:10.1016/S0140-6736(13)62222-9)
Supplement: Supplementary appendix [file mmc1.pdf]

THE LANCET

Supplementary appendix

This appendix formed part of the original submission and has been peer reviewed. We post it as supplied by the authors.

Supplement to: Field N, Mercer CH, Sonnenberg P, et al. Associations between health and sexual lifestyles in Britain: findings from the third National Survey of Sexual Attitudes and Lifestyles (Natsal-3). *Lancet* 2013; published online Nov 26. http://dx.doi.org/10.1016/S0140-6736(13)62222-9.

Web appendix: Reporting of sexual response problems (men – erectile difficulties; women – vaginal dryness) lasting at least six months in the past year in relation to demographic and health characteristics of participants who reported at least one sexual partner in the past year

	Men						Women					
	**%**	**95% CI**	**AOR[1,2]**	**95% CI**	**p value**	**Denominator[3,4]**	**%**	**95% CI**	**AOR[1,2]**	**95% CI**	**p value**	**Denominator[3,4]**
**All**	8·2%	(7·4-9·2)	-	-		4830, 5964	8·0%	(7·2-8·9)	-	-		6659, 5748
**DEMOGRAPHIC CHARACTERISTICS**												
**Age-group**					<0·0001						<0·0001	
16-24	2·4%	(1·6-3·5)	1·00			1275, 933	2·9%	(2·2-3·9)	1·00			1660, 922
25-34	3·7%	(2·8-5·0)	1·89	(1·10-3·26)		1374, 1236	4·9%	(4·0-6·1)	1·54	(1·02-2·32)		2234, 1245
35-44	4·0%	(2·7-6·0)	2·13	(1·12-4·05)		718, 1297	3·6%	(2·5-5·0)	1·06	(0·64-1·74)		1045, 1285
45-54	8·2%	(6·2-10·8)	4·23	(2·43-7·39)		630, 1186	8·8%	(6·9-11·1)	2·65	(1·71-4·12)		870, 1186
55-64	17·9%	(14·6-21·8)	9·70	(5·59-16·82)		510, 845	22·1%	(18·7-25·9)	7·57	(4·93-11·64)		569, 755
65-74	26·2%	(21·5-31·6)	16·50	(9·46-29·0)		323, 467	15·9%	(11·9-20·9)	4·87	(2·92-8·14)		281, 355
**Relationship status**					0·1234						0·0016	
Living with a partner	9·1%	(7·9-10·3)	1·00			2706, 4261	9·3%	(8·3-10·5)	1·00			3964, 4166
In a steady relationship, not cohabiting	6·1%	(4·6-8·1)	1·35	(0·94-1·94)		946, 758	5·9%	(4·5-7·7)	1·07	(0·77-1·49)		1358, 788
No steady relationship, previously cohabited	9·4%	(6·8-12·8)	1·19	(0·79-1·79)		445, 387	4·0%	(2·8-5·8)	0·47	(0·31-0·70)		749, 460
No steady relationship, never cohabited	3·8%	(2·6-5·7)	1·67	(1·02-2·74)		724, 550	2·1%	(1·1-4·1)	0·64	(0·31-1·30)		578, 329
**GENERAL HEALTH**												
**Self-reported general health status**					<0·0001						0·0111	
Very good	5·5%	(4·4-6·8)	1·00			2070, 2446	6·8%	(5·7-8·1)	1·00			2828, 2427
Good	7·8%	(6·5-9·2)	1·22	(0·90-1·66)		2046, 2604	7·3%	(6·2-8·6)	0·98	(0·75-1·29)		2846, 2417
Fair	16·3%	(13·1-20·1)	2·32	(1·60-3·37)		578, 741	12·9%	(10·3-16·0)	1·57	(1·12-2·20)		779, 708
Bad or very bad	18·8%	(12·4-27·4)	2·30	(1·30-4·07)		135, 171	14·0%	(9·4-20·5)	1·57	(0·94-2·64)		206, 195
**Long-standing illnesses or disability**					0·0019						0·0009	
None	6·0%	(5·1-7·0)	1·00			3580, 4255	6·0%	(5·2-6·9)	1·00			4836, 4020
Non-limiting	13·3%	(10·5-16·8)	1·50	(1·07-2·11)		607, 862	12·9%	(10·3-15·9)	1·70	(1·25-2·30)		844, 797
Limiting	14·5%	(11·8-17·8)	1·65	(1·20-2·26)		642, 846	12·7%	(10·4-15·5)	2·29	(1·74-3·01)		978, 930
**Number of self-reported chronic conditions[5]**					<0·0001						0·0109	
0	5·0%	(4·2-6·0)	1·00			3447, 3985	5·5%	(4·7-6·4)	1·00			4352, 3531
1	12·1%	(10·0-14·6)	1·63	(1·19-2·22)		936, 1328	11·1%	(9·2-13·3)	1·62	(1·23-2·15)		1553, 1415
>=2	20·1%	(16·3-24·5)	2·18	(1·52-3·11)		443, 647	13·8%	(11·2-16·9)	1·53	(1·10-2·12)		751, 799
**Body mass index (BMI)**					0·6876						0·1536	
Normal: 18·5-25 kg/m2	7·2%	(6·0-8·7)	1·00			2148, 2363	8·5%	(7·3-9·8)	1·00			2769, 3342
Underweight: <18·5 kg/m2	0·7%	(0·1-4·6)	-	-		78, 67	4·8%	(2·5-9·0)	1·01	(0·49-2·10)		230, 149
Overweight: 25-30 kg/m2	8·6%	(7·2-10·3)	0·93	(0·70-1·25)		1684, 2315	8·8%	(7·3-10·6)	0·79	(0·60-1·04)		1616, 1501
Obese: 30-35 kg/m2	11·3%	(8·7-14·7)	1·17	(0·80-1·73)		579, 803	8·6%	(6·4-11·7)	0·78	(0·54-1·14)		686, 656
Obese: >35 kg/m2	9·1%	(5·6-14·6)	0·92	(0·50-1·71)		178, 241	6·2%	(3·9-9·6)	0·56	(0·33-0·95)		368, 334

**Difficulty walking up stairs due to health problem**						0·0144						0·5890	
	No difficulty	7·3%	(6·4-8·3)	1·00			4467, 5451	7·4%	(6·6-8·3)	1·00			6053, 5101
	Some difficulty	16·4%	(12·1-21·9)	1·40	(0·92-2·15)		277, 393	13·6%	(10·4-17·6)	1·18	(0·83-1·67)		449, 481
	Much difficulty or unable	24·2%	(15·7-35·2)	2·10	(1·16-3·79)		86, 120	10·8%	(6·3-17·9)	0·84	(0·44-1·59)		157, 166
**SPECIFIC HEALTH CONDITIONS**													
**Any cardiac or vascular disease**[6]						0·2254						0·8819	
	No	7·6%	(6·7-8·5)	1·00			4648, 5696	7·8%	(7·1-8·7)	1·00			6541, 5612
	Yes	22·9%	(17·1-30·0)	1·34	(0·83-2·15)		181, 266	16·0%	(9·7-25·3)	1·05	(0·56-1·97)		116, 135
**Hypertension**						0·2167						0·9600	
	No	7·0%	(6·1-7·9)	1·00			4418, 5306	7·4%	(6·5-8·2)	1·00			6155, 5167
	Yes	18·6%	(14·8-23·1)	1·27	(0·87-1·84)		411, 657	14·0%	(11·0-17·8)	1·01	(0·70-1·45)		502, 580
**Diabetes**						0·2522						0·3202	
	No	7·7%	(6·8-8·6)	1·00			4662, 5701	7·9%	(7·2-8·8)	1·00			6493, 5563
	Yes	20·6%	(14·4-28·6)	1·37	(0·80-2·37)		167, 262	10·7%	(6·7-16·6)	0·73	(0·40-1·35)		164, 184
**Chronic airways disease**						0·9004						0·9317	
	No	8·2%	(7·3-9·1)	1·00			4799, 5917	8·0%	(7·2-8·9)	1·00			6629, 5721
	Yes	18·1%	(8·0-35·8)	0·94	(0·35-2·50)		30, 46	11·9%	(3·7-32·3)	1·06	(0·29-3·85)		28, 26
**Arthritis**						0·7480						0·6216	
	No	7·4%	(6·5-8·3)	1·00			4519, 5500	7·2%	(6·4-8·0)	1·00			6155, 5156
	Yes	18·5%	(14·2-23·7)	1·07	(0·70-1·65)		310, 463	15·5%	(12·3-19·4)	1·10	(0·75-1·61)		502, 591
**Broken hip or pelvis or hip replacement**						0·9467						0·0262	
	No	8·1%	(7·2-9·1)	1·00			4763, 5866	8·1%	(7·3-8·9)	1·00			6593, 5674
	Yes	16·7%	(9·0-28·9)	0·97	(0·46-2·08)		65, 95	5·0%	(1·6,-4·7)	0·25	(0·07-0·85)		65, 73
**Backache, or bone or muscle disease for >3m in past year**						0·9285						0·0004	
	No	7·8%	(6·9-8·8)	1·00			4337, 5271	7·1%	(6·3-7·9)	1·00			5848, 4994
	Yes	11·9%	(9·0-15·7)	1·02	(0·68-1·51)		491, 691	14·4%	(11·6-17·8)	1·85	(1·32-2·61)		809, 752
**Depressive symptoms**[7]						0·0016						0·0004	
	No	7·8%	(6·9-8·8)	1·00			4377, 5463	7·7%	(6·9-8·6)	1·00			5878, 5143
	Yes	12·8%	(9·5-16·9)	1·86	(1·27-2·72)		447, 494	11·0%	(8·5-14·1)	1·84	(1·31-2·58)		777, 601
**Prostate disease or surgery**						0·1463							
	No	7·7%	(6·8-8·7)	1·00			4713, 5784	-	-	-	-	-	-
	Yes	25·3%	(18·2-34·1)	1·46	(0·88-2·45)		115, 178	-	-	-	-	-	-
**Menopause**[8]												0·0200	
	No	-	-	-	-	-	-	2·3%	(1·6-3·4)	1·00			1161, 1557
	Yes	-	-	-	-	-	-	11·6%	(10·0-13·5)	1·91	(1·10-3·30)		265, 367
**SATISFACTION AND SEXUAL ACTIVITY**													
**Sexually active in the past four weeks**						0·4451						0·2429	
	No	10·9%	(8·9-13·4)	1·00			1011, 1158	10·6%	(8·8-12·7)	1·00			1408, 1245
	Yes	7·5%	(6·6-8·6)	0·89	(0·65-1·21)		3741, 4717	7·3%	(6·5-8·3)	0·85	(0·64-1·12)		5122, 4402

**Satisfied with sex life**						<0·0001						<0·0001	
	No	13·8%	(11·9-15·9)	1·00			1533, 1944	13·7%	(12·0-15·7)	1·00			2009, 1775
	Yes	5·6%	(4·7-6·6)	0·37	(0·28-0·48)		3297, 4020	5·5%	(4·7-6·4)	0·34	(0·27-0·44)		4648, 3972

[1] AOR=adjusted odds ratio.
[2] All models were adjusted for age and relationship status. Models investigating age and relationship status also adjusted for self-assessed general health status. Models investigating specific conditions were also adjusted for comorbidity, for which comorbidity was coded as 0=0–1 specific conditions and 1=≥2 specific conditions.
[3] Shows unweighted and weighted denominators, which may vary across variables due to item non-response.
[4] Denominator is participants reporting one or more sexual partner in the past year.
[5] Measure of comorbidity and includes arthritis, heart attack, coronary heart disease, angina, other forms of heart disease, hypertension, stroke, diabetes, broken hip or pelvis bone or hip replacement ever, backache lasting longer than 3 months, any other muscle or bone disease lasting longer than 3 months, depression, cancer, and any thyroid condition treated in the past year.
[6] Heart attack, coronary heart disease, angina, other forms of heart disease, and stroke.
[7] Respondents were asked whether they had often been bothered by feeling down, depressed, or hopeless in the past 2 weeks, and whether they had often been bothered by little interest or pleasure in doing things in the past 2 weeks, with a validated two-question patient health questionnaire (PHQ-2).
[8] Women deemed to be postmenopausal when they had not menstruated in the past year, with analysis restricted to those aged 45–64 years.
